# A large family of hereditary spherocytosis and a rare case of hereditary elliptocytosis with a novel SPTA1 mutation underdiagnosed in Taiwan: A case report and literature review

**DOI:** 10.1097/MD.0000000000032708

**Published:** 2023-01-27

**Authors:** Yu-Hung Shih, Ying-Chih Huang, Ching-Yeh Lin, Hsuan-Yu Lin, Su-Feng Kuo, Jen-Shiou Lin, Ming-Ching Shen

**Affiliations:** a Division of Hematology-Oncology, Changhua Christian Hospital, Changhua City, Taiwan; b Department of Research, Changhua Christian Hospital, Changhua City, Taiwan; c Department of Laboratory Medicine, Changhua Christian Hospital, Changhua City, Taiwan; d Department of Laboratory Medicine, National Taiwan University Hospital, Taipei, Taiwan.

**Keywords:** hereditary elliptocytosis, hereditary spherocytosis, RBC membrane disorders, SLC4A1, SPTA1

## Abstract

Hereditary spherocytosis (HS) has a defect in the vertically connected proteins on the cell membrane of red blood cells (RBC). Hereditary elliptocytosis (HE) has a defect in proteins that connect the cell membrane horizontally. We reported two families of RBC membrane disorders in Taiwanese, one was HS and the other was HE.

**Patient concerns:**

Case 1. A 19-year-old male student with chronic jaundice and splenomegaly. His mother, maternal uncle, grandmother, and many members of older generations also had splenomegaly and underwent splenectomy. Case 2. A 40-year-old man has experienced pallor and jaundice since the age of 20 and was found to have splenomegaly, and gall bladder stones in the older age. His younger sister also had pallor and jaundice for a long time.

**Diagnoses:**

In case 1, a peripheral blood smear showed 20% spherocytes. Eosin-5-maleimide labeled RBC by flow cytometry showed a result of 30.6 MCF (cutoff value: 45.5 MCF). He was diagnosed with HS. The gene analysis identified a heterozygous mutation with c.166A > G (p.Lys56Glu) in the SLC4A1 gene in this proband, his mother, and maternal uncle. In case 2, more than 40% of ellipsoid RBC present in the peripheral blood smear. He was diagnosed with HE. Genetic analysis of the SPTA1 gene identified a novel heterozygous exon2, c.86A > C, p.Gln29Prol mutation.

**Interventions:**

The two patients had compensated anemia, clinical follow-up instead of splenectomy was done.

**Outcomes:**

The two patients had normal daily activities and lives.

**Lessons:**

We reported two Taiwanese families, one was hereditary spherocytosis affected by a heterozygous mutation with c.166A > G (p.Lys56Glu) in SLC4A1, and the other was hereditary elliptocytosis caused by a novel heterozygous SPTA1 gene mutation, c. 86A > C, p.Gln29Prol. These 2 seemingly common hereditary red blood cell membrane protein defects induced by hemolysis are usually underdiagnosed or misdiagnosed.

## 1. Introduction

Normal erythrocytes have a lipid bilayer membrane with proteins that adhere to the cytoskeleton and confer a normal biconcave shape, providing optimal surface/volume ratio, maintaining structural integrity, and adequate ion permeability.^[1]^ Red blood cell membrane disorders are a heterogeneous group of inherited disorders caused by certain mutations in genes that encode proteins that link the membrane cytoskeleton to the lipid bilayer. Deficiency or dysfunction of these proteins leads to reduced deformability of red blood cell (RBC) in the splenic trapping and narrow capillary beds, which causes hemolysis. In addition, defects in some membrane proteins have abnormal ionic permeability, with an increasing influx of sodium into the intracellular space. RBC membrane disorders can be categorized as altered membrane structural organization (hereditary spherocytosis and hereditary elliptocytosis) and altered membrane transport function (hereditary stomatocytosis and hereditary xerocytosis).^[2,3]^ Among these diseases, hereditary spherocytosis (HS) and hereditary elliptocytosis (HE) are the most common types in the world.^[2–8]^

HS is a defect in the vertically connected proteins on the cell membrane of red blood cells.^[2–5]^ HS is widely distributed around the world, especially in the Caucasian ethnic group.^[8–10]^ HE, is a defect in proteins that connect the cell membrane horizontally.^[2–5]^ A distribution of HE has obvious regional characteristics. The prevalence of HE, especially the Southeast Asian ovalocytosis (SAO) subtype, is higher in malaria-endemic areas.^[11]^

In the study, we reported 2 families of RBC membrane disorders in Taiwanese, 1 was HS and the other was HE. In the case of HS, the genetic mutation (c.166A > G,p.Lys56Glu in the SLC4A1) was previously published,^[12]^ while in the case of HE, a novel mutation was newly discovered. We provided detailed personal and family history, physical examinations, and laboratory findings for these 2 underdiagnosed disorders to raise awareness among physicians and reviewed related literature.

## 2. Case presentation

### 2.1. Patients and methods

In this investigation, 1 patient was diagnosed with HS and the other with HE at Changhua Christian Hospital. Hemolytic anemia, elevated bilirubin, splenomegaly, spherocytes or elliptocytes in the peripheral blood smear, and family history were used to diagnose HS and HE. Both patients were tested for osmotic fragility, flow cytometry of eosin-5-maleimide labeled red blood cell (EMA-labeled RBC), and genetic analysis using next generation sequencing (NGS) and polymerase chain reaction. Following the manufacturer’s instructions, NGS sequencing libraries were created using the Agilent SureSelect Human All Exon V6 kit (Agilent Technologies, CA). The libraries were sequenced on an Illumina NovaSeq 6000 platform, and Genomics BioSci& Tech Co. generated 150 bp paired-end reads. The variations of RBC membrane diseases candidate genes were chosen for Sanger sequencing, and the amplified product was sequenced according to the manufacturer’s instructions using an automated DNA sequencing analyzer, ABI3130xl (Applied Biosystems, Foster City, CA). All patients gave their informed consent to participate in this study, which was carried out in accordance with the Declaration of Helsinki and approved by our institution’s Ethics Committee.

### 2.2. Case 1

The proband was a 19-year-old male student with chronic jaundice and mild splenomegaly. He had a history of neonatal hyperbilirubinemia (total bilirubin of 19.72 mg/dL, direct bilirubin of 1.31 mg/dL at birth), which was relieved by phototherapy. He has had no notable symptoms or signs except that hyperbilirubinemia and mild splenomegaly were persistently detected on several occasions during the health examination at high school. His mother also had splenomegaly and suffered from abdominal pain and pallor at the age of 10. She received a splenectomy 1 year later. The patient’s maternal uncle, grandmother, and many members of older generations also had splenomegaly and underwent splenectomy. His parents did not pay attention to his symptoms until January 2018, when he was brought to our clinic to seek a possible cause.

**Figure 1.**
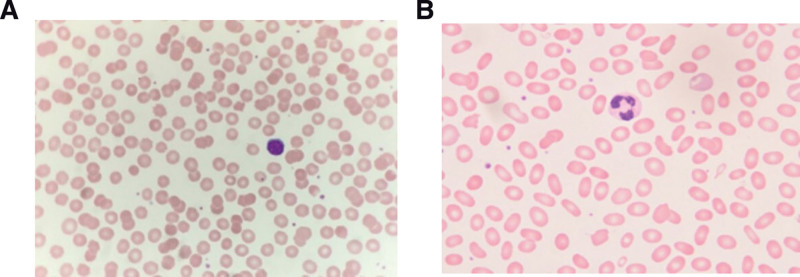
**Representation of peripheral blood smear.** (A) Spherocytosis in the case 1 with HS. (B) Elliptocytosis in the case 2 with HE.

**Figure 2.**
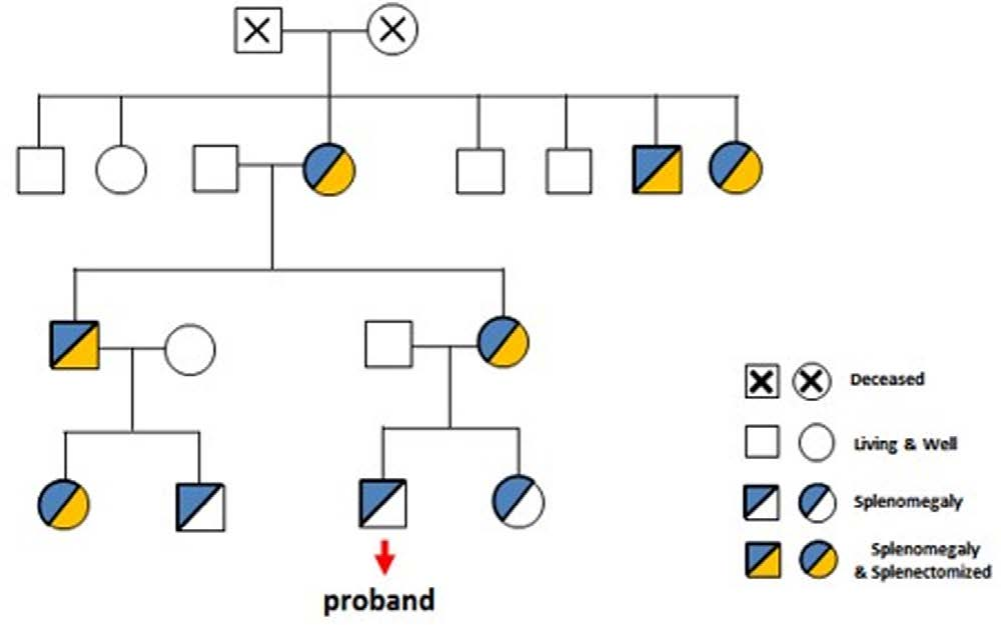
**Pedigree of the Family.** Splenomegaly was found in 9 family members and 6 of these members were splenectomized.

On physical examination, he was well developed and without a pale face. But he was found to have icteric sclera and mild splenomegaly. Laboratory tests revealed that WBC was 11,000/uL, platelet was 3,11,000/uL, Hb was 14.9 gm/dL, MCV was 88.2 fl, MCHC was 37%, white blood cell classification was normal, total bilirubin was 5.26 mg/dL, direct bilirubin was 0.33 mg/dL, GOT was 17 IU/L, GPT was 7 IU/L, LDH was 164 U/L, haptoglobin was < 8.0 mg/dL, and Coombs’ test was negative. A peripheral blood smear showed 20% anisocytosis and 20% spherocytes (Fig. 1). The analysis of EMA-labeled RBC by flow cytometry showed a result of 30.6 MCF (mean channel fluorescence). The cutoff value is 45.5 MCF. Tracing the family pedigree, splenomegaly was found in 9 family members, and 6 of these members were splenectomized (Fig. 2). The gene analysis identified a heterozygous mutation with c.166A > G (p.Lys56Glu) in the SLC4A1 gene in this proband, his mother, and maternal uncle. This mutation has been reported previously.^[12]^

### 2.3. Case 2

A 40-year-old man native to Taiwan has experienced mild pallor and jaundice since the age of 20. He had no symptoms in his usual life. He suffered from aggravated epigastric pain with clay-colored stool for days in August 2019, at the age of 39.

Physically, he was found to be slightly anemic and icteric. The spleen was barely palpable. Laboratory examinations revealed that Hb was 11.7 gm/dL, WBC was 7500/uL, platelet count was 320,000/uL, MCV was 84.1 fl, MCH was 58.7 pg, MCHC was 34.2 %, RDW was 19.1%, and white cell classification was normal. There were more than 40% of ellipsoid RBCs present in the peripheral blood smear (Fig. 1). The biochemical test revealed that total bilirubin was 14 mg/dL, direct bilirubin 7.7 mg/dL, GOT 314 IU/L, and GPT 482 IU/L. The abdominal sonography showed mild splenomegaly, multiple gall bladder stones, and bilateral intrahepatic duct dilatation. Endoscopic retrograde cholangioscopic removal of gall stones was successfully carried out. Three weeks later, obstructive jaundice and hepatitis had subsided, and total bilirubin was 3.9 mg/dL, direct bilirubin was 1.03 mg/dL, GOT was 29 IU/L, and GPT was 54 IU/L. EMA-labeled RBC by flow cytometry showed a negative result. The osmotic fragility test was within normal limits. Genetic analysis of the SPTA1 gene identified a novel heterozygous exon2, c.86A > C, p.Gln29Prol mutation. Family history revealed that his younger sister also had pallor and jaundice for a long time.

## 3. Literature review and discussion

### 3.1. Pathophysiology and genetic variations

HS is an inherited disorder caused by certain mutations in genes that encode proteins that vertically link the membrane cytoskeleton to the lipid bilayer. Deficiency or dysfunction of these proteins leads to microvesiculation and progressive membrane loss. Loss of membrane reduces the ratio of RBC surface area to volume, which in turn creates progressively more spherical cells.^[8,10]^

Table 1 summarizes the variant genes described in the HS, which can be spectrin-related SPTA1 and SPTB genes, respectively, ankyrin-related ANK1 gene, band 3 related SLC4A1 gene, and band 4.2 related EPB42 gene. Three-fourths of HS mutations are autosomal dominant (AD) inheritance, as observed in the family we described. These dominant mutations include SPTB, ANK1, and SLC4A1 mutations. However, autosomal recessive transmission can occur, particularly with SPTA1 and EPB42 mutations. De novo mutations are also described.^[9]^

**Table 1:** Molecular and genetic characteristics of hereditary spherocytosis.

Gene	Membrane protein	Patients with HS	Prevalent mutation	Protein deficiency	Hereditary	Disease severity
ANK1	Ankyrin	USA and Europe 40%–65%, South Korea 52%, Japan 5%–10%, China 50%	Frameshift, nonsense, missense, abnormal splicing, promoter region	Ankyrin, Ankyrin/spectrin	AD, AR, de novo	Mild-moderate
SLC4A1	Band 3	20%–35%	Missense, nonsense/frameshift, larger mutant protein, polymorphism	Band 3	AD	Mild-moderate
EBP42	Protein 4.2	USA and Europe < 5%, Japan 45%–50%	Missense, nonsense or deletion, splicing	Protein 4.2	AR	Mild-moderate
SPTA1	α spectrin	<5%	Splicing/skipping Spα^LEPRA^allele	α spectrin, α/β spectrin	AR	Severe
SPTB	β spectrin	15%–30%,China 30%	Null mutations, nonsense or in non-coding sequence, missense, polymorphism	β spectrin	AD, de novo	Mild-moderate

Data modified from Perrotta et al^[8]^ and Bolton-Maggs et al^[9]^

AD = autosomal dominant, AR = autosomal recessive.

As for HE, there is a problem with proteins that connect the cell membrane horizontally, which makes red blood cells repeatedly shaped into ellipsoids during the process of RBCs passing through narrow capillary beds. HE can be classified into common HE, hereditary pyropoikilocytosis (HPP), and SAO according to its phenotype. Most cases are a common type of hereditary elliptocytosis, and more than 90% of inherited genes are heterozygous. These people have no clinical symptoms or only mild hemolytic anemia, while HPP, occurring in homozygous or compound heterozygous patients, accounts for < 10% of HE, will suffer from moderate to severe hemolytic anemia and need regular blood transfusions.^[13]^

In HE, 65% of the cases have mutations in the SPTA1 gene, resulting in defects in alpha spectrin structure and function. 30% of the cases have mutations in the SPTB gene. The remaining 5% of HE is contributed to by EPB41 genetic mutations that regulate protein 4.1.^[2]^ Most of the inherited forms are AD, and only the most serious HPP types are inherited by autosomal recessive. In addition, SAO is prevalent in Southeast Asia. The main causes are band 3 defects, and its related mutant gene comes from SLC4A1 deletion of 27 base pairs that lead to the loss of 9 amino acids, which is also AD inherited (Table 2).^[2]^

**Table 2:** Phenotype, prevalence, and genetic characteristics of hereditary elliptocytosis.

Phenotype	Distribution/prevalence	Gene	Membrane protein	Hereditary	Disease severity
HE	Africa, Mediterranean, or Southeast Asia: 1%–2%	EPB41 (5%)	protein 4.1	AD	Heterozygous: none to mild Homozygous or compound heterozygous: moderate to severe
		SPTA1 (65%)	α spectrin	AD	
		SPTB (30%)	β spectrin	AD	
HPP		SPTA1	α spectrin	AR	Severe
SAO	Southeast Asia: 5%–25%	SLC4A1	Band 3	AD	None to mild

HE = hereditary elliptocytosis, HPP = hereditary pyropoikilocytosis, SAO = southeast asian ovalocytosis, AD = autosomal dominant, AR = autosomal recessive.

### 3.2. Epidemiology

HS is widely distributed around the world, especially in the Caucasian ethnic group. The prevalence rate can reach 1/2000 in North America and Northern Europe.^[8–10]^ In different countries or regions, the distribution frequency of its gene variation also has its own characteristics. In the United States and Europe, ANK1 mutations account for 40% to 65% of all HS patients; in Japan, EPB42 mutations account for 40% to 50% of all HS^[8]^; and in South Korea, the majority of HS patients (52%) have ANK1 mutations(Table 1).^[14]^ In China, the estimated prevalence of HS is 1.27/100 000 in males and 1.49/100 000 in females.^[15]^ In studies, ANK1 mutations (about 50%) are most commonly present, followed by SPTB mutations (about 30%).^[16–21]^ But some investigations reported that Chinese HS patients are affected by band 3 deficiency. Because of the small number of reported cases, it is necessary to collect more data to determine the actual rates of erythrocyte membrane protein deficiency in Chinese HS patients.^[22]^ The latest report by Keiko Shimojima Yamamoto et al from NGS analysis of 13 Japanese with hereditary spherocytosis demonstrated that ANK1 variants were found to be the most common, being found in 46% (6/13) of the patients and followed by SPTB variants identified in 31% (4/13) of the patients. This distribution of the variants was similar to those observed in other Asian countries but was different from those observed in non-Asian countries. The distribution of HS-related variants was also different from that identified in a previous study on Japanese patients with HS.^[23]^ No epidemiological data for HS in Taiwan has been reported.

The true prevalence of HE is unknown because many mildly affected individuals are likely to remain undiagnosed. The global prevalence has been estimated to be 1 in every 2000 to 4000 people (0.05%–0.025%).^[5]^

HE is most common in individuals of African, Mediterranean, or Southeast Asian descent. It has been reported that in areas such as West Africa, the prevalence of HE is as high as 1% to 2%. In particular, SAO is a very common condition in the aboriginal peoples of Papua New Guinea, Indonesia, Malaysia, the Philippines, and southern Thailand, with a prevalence varying between 5% and 25%.^[24–26]^ SAO also confers resistance against Plasmodium falciparum infection, likely because of alterations in band 3, which is one of the malaria receptors. Generally, SAO individuals are asymptomatic. Due to its high prevalence in Southeast Asia, SAO is not a disease, but rather an erythrocyte polymorphism.^[3]^ In vitro, Plasmodium falciparum infection in elliptocytes carrying such spectrin alleles revealed that these alleles are genetic causes of malaria resistance. However, several spectrin mutations or polymorphisms have yet to be demonstrated in vivo as new drivers of innate resistance to malaria (11).

In Korea, HE is the cause of 1.4% of cases of hereditary hemolytic anemia.^[27]^ In Japan, HE accounts for 13.5% of hereditary red cell membrane disorders.^[28]^ No epidemiological data on HE has been published in China. There are only a few cases and a few series of reports concerning Chinese hereditary elliptocytosis.^[29–31]^ No cases have been reported from Taiwan.

### 3.3. Manifestations and complications

Typical manifestations of HS or HE include hemolysis with anemia, jaundice, reticulocytosis, gallstones, splenomegaly, spherocytes, or elliptocytes on the peripheral blood smear, and a positive family history of the disease. Some patients with HS may present with hematologic crises, which may be hemolytic, aplastic, or megaloblastic. The aplastic crisis is associated with parvovirus B19 infection. Hemoglobin and reticulocytes will decrease first, and then the erythroblasts in the bone marrow will also decrease.^[32–34]^ Megaloblastic crises are caused by folate deficiency and can be corrected by adequate folate supplementation. Extramedullary hemopoietic tumors attributed to the ineffective hematopoiesis of hereditary spherocytosis have been described in many case reports. Other complications, such as growth failure and leg ulcers, are also reported.^[35,36]^

### 3.4. Diagnosis and laboratory findings

Several tests are available to confirm the diagnosis of HS and HE. But these tests can be omitted if classic findings (hemolysis, jaundice, splenomegaly, spherocytosis) and positive family history have been identified.^[2,9,37]^

EMA (eosin-5-maleimide) is an eosin-based fluorescent dye that binds to RBC membrane proteins, especially band 3 and Rh-related proteins.^[38]^ The mean fluorescence of EMA-labeled RBCs from individuals with HS is lower than controls, and this reduction in fluorescence can be detected in a flow cytometry-based assay. EMA is now a standard test for HS screening but has less efficacy in the case of HE.^[4]^ The osmotic fragility test (OFT) was conducted according to the fraction of hemoglobin released in hypotonic salt solutions of various osmolarities.^[39]^ OFT is an old method for detecting RBC membrane disorders, and it has a certain value in the diagnosis of HS, although the sensitivity is only 60% to 70%. As for HE, there will be no abnormalities of OFT in common HE and SAO except HPP and more severe HE in the OFT. In our 2 cases, case 1 carried HS, presented a low EMA level, and a positive finding of OFT, but case 2 carried HE in the normal range of these tests.

The glycerol lysis test (GLT) and the acidified GLT (AGLT) are modifications of the OFT that add glycerol (in the GLT) or glycerol plus sodium phosphate (to lower the pH to 6.85, in the AGLT) to the hypotonic buffered salt solutions.^[40,41]^ The sensitivities of these tests were evaluated in a 2012 study that tested samples from 150 individuals known to have HS.^[42]^ The results of sensitivity were as follows: 93% of EMA bindings, 68% of fresh osmotic fragility, 81% of incubated osmotic fragility, 95% of AGLT, and 61% of GLT.

Osmotic gradient ektacytometry measures RBC deformability under defined shear stress as a function of suspended medium osmolality. The test is used to screen for inherited RBC membrane disorders such as HS, HPP, SAO, and hereditary stomatocytosis.^[4,39]^ However, the technique of osmotic gradient ektacytometry has not been evaluated in formulating the guidelines because of a lack of its general availability.

Quantification of the amount of spectrin, ankyrin-1, band-3 protein, or protein 4.2 in the erythrocyte membrane can support the diagnosis of these diseases. However, some patients might have only a 10% to 15% reduction in the affected protein. The simplest method is SDS-PAGE of red blood cell membranes, but this technique reveals abnormalities in only 70% to 80% of patients.^[43]^

Polymerase chain reaction-based mutation screening techniques have been applied to the detection of HS and HE-associated mutations. Next-generation gene sequencing can also be used in transfusion-dependent cases in which the RBC phenotype cannot be evaluated.

## 4. Discussion

In Taiwan, cases of HS are occasionally encountered in clinical practice, but few associated studies have been published. The actual prevalence of HS in Taiwan is unknown.

The family of Case 1 is a typical presentation of HS inheritance. Nine members of the family had splenomegaly, and 6 of them had undergone splenectomy. In a sense, we can mitigate HS by splenectomy. However, splenectomy is reserved for HS patients who have suffered moderate to severe hemolysis. Spleen size is not an absolute indication of splenectomy. Many people in this family have been overtreated, and the exact etiology of splenomegaly in these people has not been determined yet.

In Case 1, obvious jaundice was found when he was born. We should take inherited hemolytic disease into consideration, especially unconjugated jaundice, which is predominant in the period of infancy. Although most cases of mild HS do not have major complications, it is a fact that the phenomenon of case 1 has been selectively neglected by physicians over the years. Notably, we found a heterozygous mutation with c.166A > G (p.Lys56Glu) in the SLC4A1 gene in case 1. We herein highlight that congenital hemolysis must be taken into diagnosis in the case of anemia, jaundice, splenomegaly, and family history. Physicians should be aware of inherited hemolytic diseases, especially HS, which is an AD inheritance prevalent.

Cases of HE are rarely reported by Taiwanese, and no 1 has investigated the epidemiology of HE in Taiwan. As mentioned above, the endemic areas of HE are parallel to malaria-endemic areas. HE is prevalent in the prevalence of malaria. Southeast Asia is an area where malaria is endemic, so the prevalence rate is high, but northeast Asia, such as South Korea and Japan, has a low rate of prevalence. Taiwan is located at the junction of Northeast Asia and Southeast Asia, and its climate and environment are also in between. More than 90% of the Taiwanese ethnic group are Han people from mainland China, and only a few are aboriginal people. As a result of the intermarriage of the Han and the aboriginal people over the past 3 to 4 hundred years, Han and the aboriginal peoples may both carry each other’s genes. Therefore, needs more study to determine the origin of this variant gene carried by case 2. By the way, there was a study that investigated the evolutionary origins of Southeast Asian ovalocytosis, and the aborigines of Taiwan were also included in the Austronesian language group. However, the results of this report indicated that no causal mutation in the SLC4A1 gene has been found in the aborigines of Taiwan.^[44]^

The development of Case 2 was not affected by congenital hemolytic diseases. But in adulthood, he suffered from recurrent cholecystitis and cholangitis due to gallstones caused by hemolysis. Because of this, HE was discovered to have a novel mutation with heterozygous exon2, c.A86C, p.Gln29Prol mutation of the SPTA1 gene was found in case 2 of our study.

## 5. Conclusion

We reported 2 Taiwanese families, 1 was hereditary spherocytosis affected by a heterozygous mutation with c.166A > G (p.Lys56Glu) in SLC4A1 (Band 3 protein), and the other was hereditary elliptocytosis caused by a novel heterozygous SPTA1 gene mutation, c. 86A > C, p.Gln29Prol. There were no similar case series reports in Taiwan. These 2 seemingly common hereditary red blood cell membrane protein defects induced by hemolysis are usually underdiagnosed or misdiagnosed. We highlighted these inherited hemolytic diseases herein to enhance the awareness of physicians and provide a literature review. We also had an interesting finding that Taiwan aborigines do not carry the SLC4A1 deletion, which exists in SAO and is popular in Southeast Asian people, although Taiwan aborigines belong to the Austronesian language family.

## Author contributions

**Conceptualization:** Ming-Ching Shen.

**Formal analysis:** Ying-Chih Huang.

**Investigation:** Yu-Hung Shih, Ying-Chih Huang.

**Methodology:** Ying-Chih Huang.

**Resources:** Ching-Yeh Lin, Hsuan-Yu Lin, Su-Feng Kuo, Jen-Shiou Lin.

**Software:** Ying-Chih Huang.

**Supervision:** Ming-Ching Shen.

**Writing – original draft:** Yu-Hung Shih.

**Writing – review & editing:** Ming-Ching Shen.
